# Clinical and quality-of-life outcomes of a combined synthetic scaffold and autogenous tissue graft procedure for articular cartilage repair in the knee

**DOI:** 10.1186/s13018-022-03010-x

**Published:** 2022-02-20

**Authors:** Fernando Martins Rosa, Julio Cesar Fernandes, Josée Delisle, Pierre Ranger, Mauro Batista Albano, Edmar Stieven Filho

**Affiliations:** 1 Graduate Program in Clinical Surgery, Hospital de Clínicas, Universidade Federal do Paraná (UFPR), R. Gen. Carneiro, 181, Curitiba, PR Brazil; 2 Orthopaedics and Traumatology Department, Université de Montréal, Montreal, QC Canada; 3 Orthopaedics and Traumatology Department, CIUSSS Nord de L’Ile de Montréal, Hôpital du Sacré-Coeur de Montréal, 5400 Boul Gouin O, Montreal, QC H4J 1C5 Canada; 4 Hospital de Clínicas, UFPR, R. Gen. Carneiro, 181, Curitiba, PR Brazil; 5 Orthopaedics and Traumatology Department, Hospital de Clínicas, UFPR, R. Gen. Carneiro, 181, Curitiba, PR Brazil; 6 Rua Guaianazes, 919, Curitiba, PR 80320-240 Brazil

**Keywords:** Cartilage, Tissues, Support, Cartilage, Hyaline, Orthopedics, Arthroplasty, Subchondral, Knee surgery, Sports surgery, Scaffold

## Abstract

**Background:**

Injuries to the articular cartilage of the knee often fail to heal properly due to the hypocellular and avascular nature of this tissue. Subsequent disability can limit participation in sports and decrease quality of life. Subchondral bone perforations are used for the treatment of small defects. Filling out the central portion in larger lesions becomes difficult, and scaffolds can be used as adjuvants, providing a matrix onto which the defect can be filled in completely. Also, autogenous cartilage grafts can be combined, acting as an inducer and improving healing quality, all in a single procedure.

**Methods:**

This observational study evaluated the clinical and quality-of-life outcomes of patients with articular cartilage lesions of the knee undergoing repair via a microfracture technique combined with a synthetic scaffold and autogenous cartilage graft, with transosseous sutures and fibrin glue fixation, at 12 months of follow-up. Secondarily, it assessed whether combined procedures, previous surgical intervention, traumatic aetiology, lesion location, and age affect outcomes. The sample consisted of adult patients (age 18–66 years) with symptoms consistent with chondral or osteochondral lesions, isolated or multiple, ICRS grade III/IV, 2–12 cm^2^ in size. Patients with corrected angular deviations or instabilities were included. Those with BMI > 40 kg/m^2^, prior total or subtotal (> 30%) meniscectomy, second-look procedures, and follow-up < 6 months were excluded. Pain (VAS), physical activity (IKDC), osteoarthritis (WOMAC), and general quality of life (SF-36) were assessed.

**Results:**

64 procedures were included, comprising 60 patients. There was significant improvement (*P* < 0.05) in VAS score (5.92–2.37), IKDC score (33.44–56.33), and modified WOMAC score (53.26–75.93) after surgery. The SF-36 showed significant improvements in the physical and mental domains (30.49–40.23 and 46.43–49.84 respectively; both *P* < 0.05).

**Conclusions:**

Combination of microfractures, autogenous crushed cartilage graft, synthetic scaffold, and transosseous sutures with fibrin glue provides secure fixation for treatment of articular cartilage lesions of the knee. At 12-month follow-up, function had improved by 20 points on the IKDC and WOMAC, and quality of life, by 10 points on the SF-36. Age > 45 years had a negative impact on outcomes.

## Background

Injuries to the articular cartilage of the knee often fail to heal properly due to the hypocellular and avascular nature of this tissue. Subsequent disability can limit participation in sports and decrease quality of life, making surgical treatment of these lesions an attractive option [1]. In a meta-analysis, surgical repair of articular cartilage lesions was associated with a 76% rate of return to sport. Medium-term activity scores for this procedure were comparable to those achieved with meniscus repair [2].

The subchondral bone perforations introduced by Pridie [3] and modified by Steadman et al. [4] are still used for the treatment of small (2–3 cm^2^), International Cartilage Repair Society (ICRS) grade III and IV chondral defects. They can repair the defect from the periphery towards the centre, but in lesions larger than 3 cm^2^, filling out the central portion becomes difficult. To improve this, scaffolds can be used as adjuvants, providing a matrix onto which the defect can be filled in completely [5]. Autogenous cartilage grafts can be combined with this matrix, acting as an inducer and improving healing quality, all in a single procedure [6].

Studies have reported the impact of surgical cartilage repair on physical activity scores (International Knee Documentation Committee, IKDC), osteoarthritis scales (Western Ontario and McMaster Universities Arthritis Index, WOMAC), and visual analogue scales (VAS) of pain; however, few have assessed the impact on quality-of-life outcomes measured with validated instruments, such as the Medical Outcomes Short-Form Health Survey (SF-36). A brief review of the literature found no studies using the WOMAC, IKDC, and SF-36 concurrently.

Thus, the objective of this study was to evaluate the clinical and quality-of-life outcomes of the microfracture technique, combined with autologous cartilage graft and a synthetic matrix, in the repair of articular cartilage lesions in the knee.

## Methods

This was a retrospective analysis of medical records of patients who underwent surgical repair of articular cartilage lesions at a single hospital. Approval was obtained from the Ethics Committee of University of Montreal (dossier no. CER 2021-2115).

The inclusion criteria were patients aged 18–66 years with symptoms consistent with single or multiple chondral or osteochondral lesions, grade III/IV on the International Cartilage Repair Society (ICRS) classification, 2–12 cm^2^ in size.

The exclusion criteria were body mass index (BMI) > 40 kg/m^2^, meniscectomy > 30% in the treated compartment, second-look procedures, and duration of follow-up 6 months or less.

Patients with axis deviation of the lower limbs, patellofemoral joint abnormalities, and ligament instability were accepted. Any such issues were corrected during the same surgical procedure.

Data were collected pre-operatively and at 6 and 12 months post-operatively. All patients completed a subjective VAS for pain; the transformed WOMAC score and the IKDC scale for functional assessment; and the SF-36 instrument for quality of life.

Patients were divided into two groups for comparison, in order to ascertain whether there was any statistically significant difference in the following variables at 12 months versus baseline: combination with another intervention during the same procedure; history of previous surgery; aetiology (traumatic or non-traumatic); lesion location (patellofemoral or femorotibial); and age (older or younger than 45 years).

Failure was defined as implant loosening, absence of improvement within 12 months, or need for a second procedure.

The scaffold used was a synthetic, single-phase, sterile, acellular, absorbable sponge-type membrane, 1.8 mm thick, composed of hyaluronic acid and poly-glycolic acid (Chondrotissue®, Biotissue AG, Zürich, Switzerland).

### Surgical procedure

Patients were placed in the supine position with the knee at 90°. No tourniquet was used. Arthroscopy was performed to confirm the size and severity of the cartilage lesion, followed by a mini-arthrotomy depending on the location of the lesion.

The subchondral bone was exposed until a 90-degree well-defined border between the cartilage and the bone. Microfractures were performed from the periphery towards the centre, with a diameter of 1.2 mm and a distance of 3–4 mm between drill holes.

A piece of disposable foil was placed on the bed of the lesion to mark out its shape. This was then used as a mould for the membrane, which was cut to fit the defect.

**Fig. 1:**
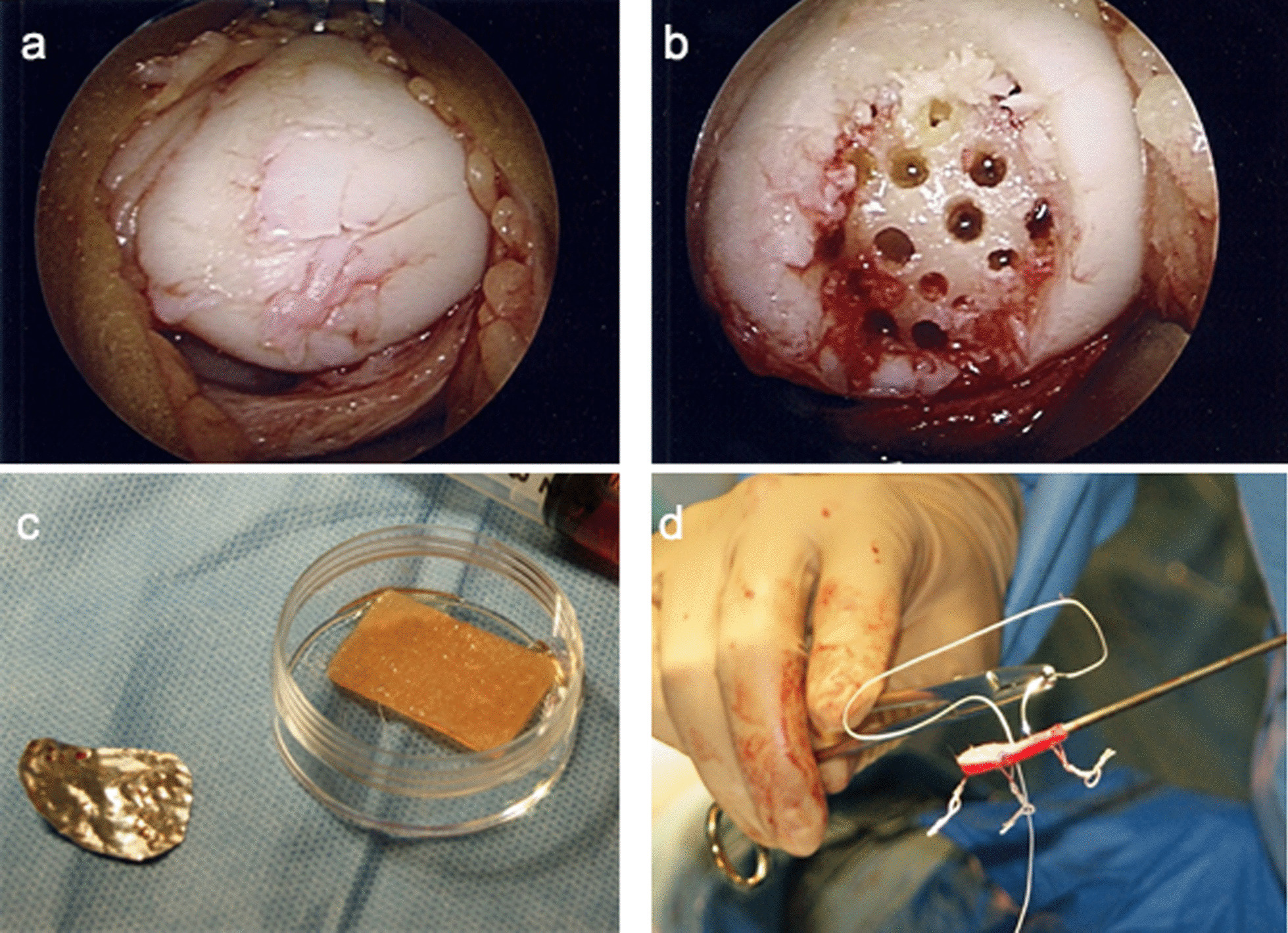
**a** Grade IV patella lesion. **b** Microfractures. **c** Foil mould, cut to shape. **d** Scaffold sutures

The scaffold was prepared with sutures (#1 absorbable poly-glycolic acid) at each end of the membrane. The suture knots were pre-formed so they would remain buried in the bone. The scaffold was hydrated with the patient’s own blood (Fig. 1).

**Fig. 2:**
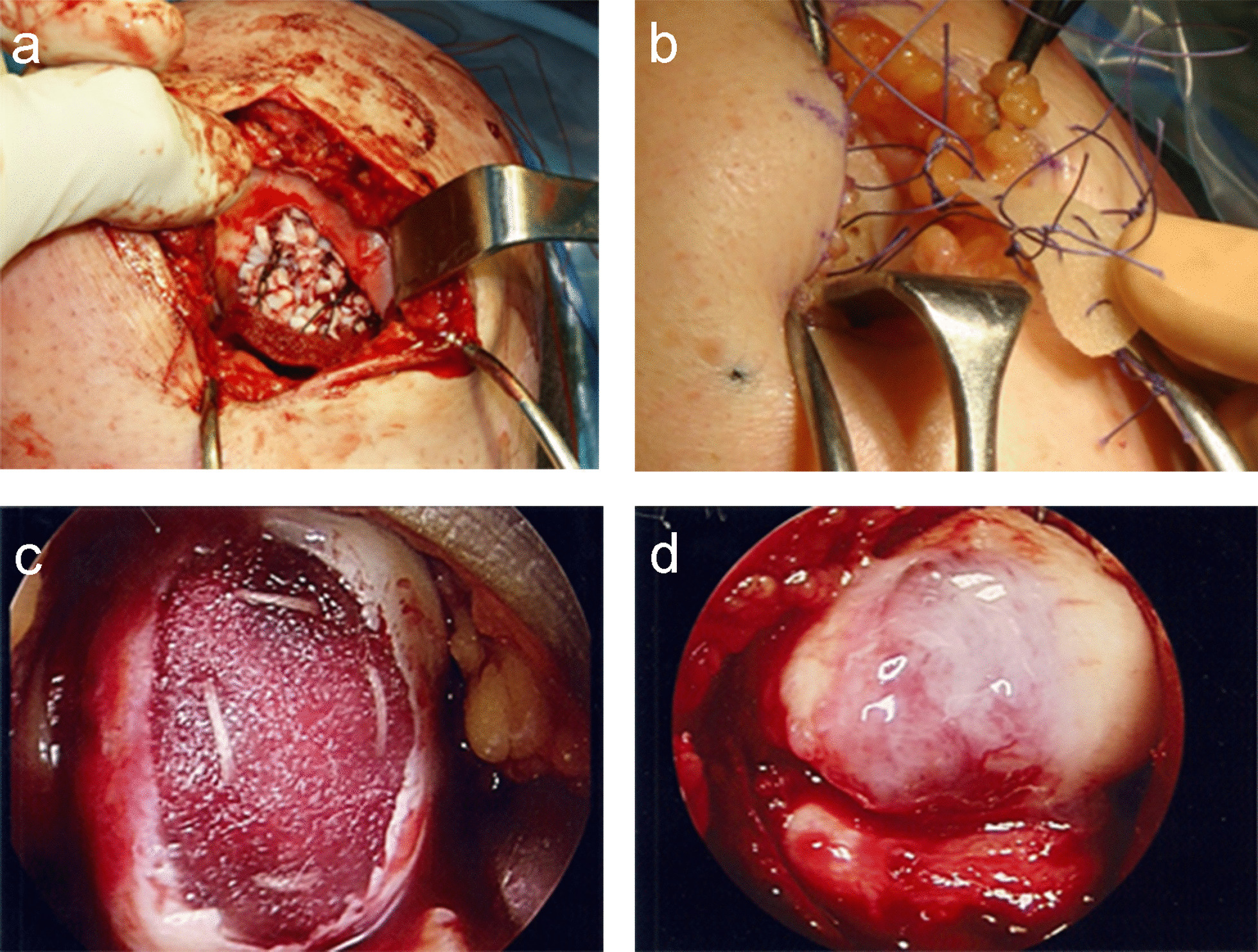
**a** Crushed autogenous cartilage. **b** Scaffold with transosseous sutures. **c** Scaffold in place. **d** Final appearance

Samples of autogenous cartilage from the healthy intercondylar area were harvested with a trephine and crushed with a scalpel blade. The resulting autogenous cartilage graft was placed in the bed of the lesion, followed by the scaffold, and the membrane was secured with buried transosseous sutures. After membrane fixation, fibrin glue was applied for added final stability (Fig. 2).

No orthotic devices or splints were used post-operatively. In cases where any additional procedures were necessary, such as osteotomies or repair of ligament injuries, these were performed before the articular surface was repaired.

Rehabilitation was started on the first postoperative day, with range-of-motion exercises. Patients were cleared for progressive weight-bearing immediately in case of patellofemoral lesions and after the sixth postoperative week for femoral condyle lesions. Activities were resumed 12 weeks after the operation, and gradual return to sport was allowed after 6 months.

### Statistical analysis

Patients were evaluated at three separate time points: pre-operatively, and at 6 and 12 months post-operatively. Repeated-measures analysis of variance (ANOVA) was performed to identify significant differences between time points. When comparing VAS, WOMAC, IKDC, and SF-36 scores at the last time point between subgroups, analysis of covariance (ANCOVA) was used, adjusting for patient’s pre-operative scores. P-values < 0.05 were deemed statistically significant.

## Results

Sixty patients were evaluated (36 women and 24 men, mean age 44 ± 12.7 years), for a total of 64 knees (33 right and 31 left).

Overall, 75 articular cartilage lesions were treated (25.3% grade III and 74.6% grade IV). Eleven knees had two lesions each. The average lesion size was 4.4 ± 2 cm^2^; the smallest lesion was 2 cm^2^, and the largest, 12 cm^2^. The patella was the most frequent site of injury, accounting for 39.1% of the cases, followed by the femoral condyles with 37.5%. The remainder were combined lesions.

When a concomitant procedure was combined with articular surface repair, osteotomy of the anterior tibial tuberosity (ATT) was the most common intervention, prevalent in 23.4% of cases (always performed to correct patellar alignment); 14.1% had a partial meniscectomy, 14.1% had a tibial valgus osteotomy, and 18.7% had no additional procedures.

Just over half of procedures (51.6%) were performed on patients with progressive lesions and no preceding trauma, while the remaining 48.4% had a history of trauma. In 71.9% of cases, there had been no previous surgery.

Regarding pain improvement, repeated-measures ANOVA found VAS scores significantly decreased from 5.92 (± 2.2) pre-operatively to 2.37 (± 1.9) (*P* < 0.001) at 12 months postoperatively.

**Fig. 3:**
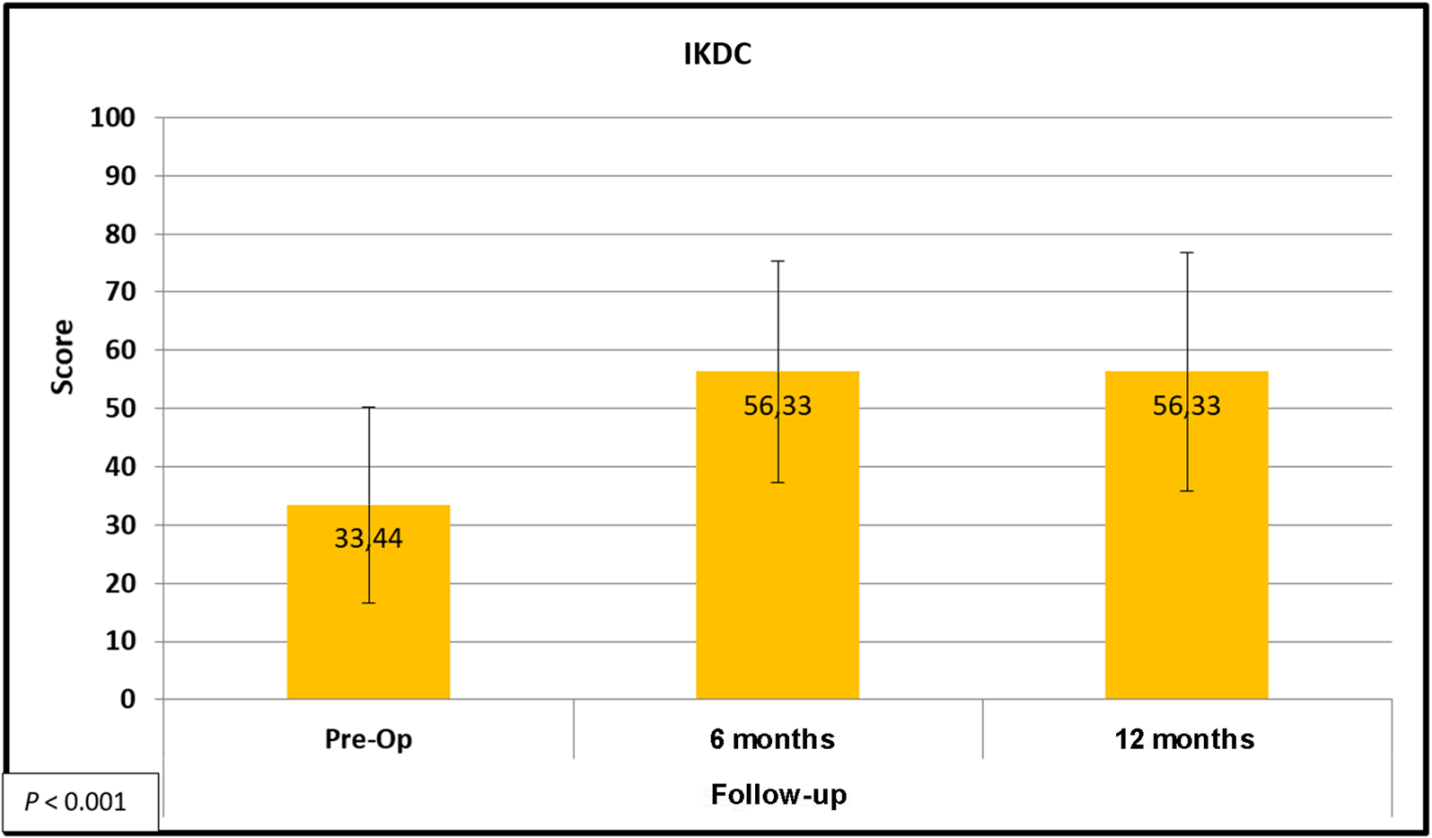
Descriptive clinical outcomes of articular joint repair: IKDC score

IKDC scores were consistent with progressive functional improvement, as shown in Fig. 3.

**Fig. 4:**
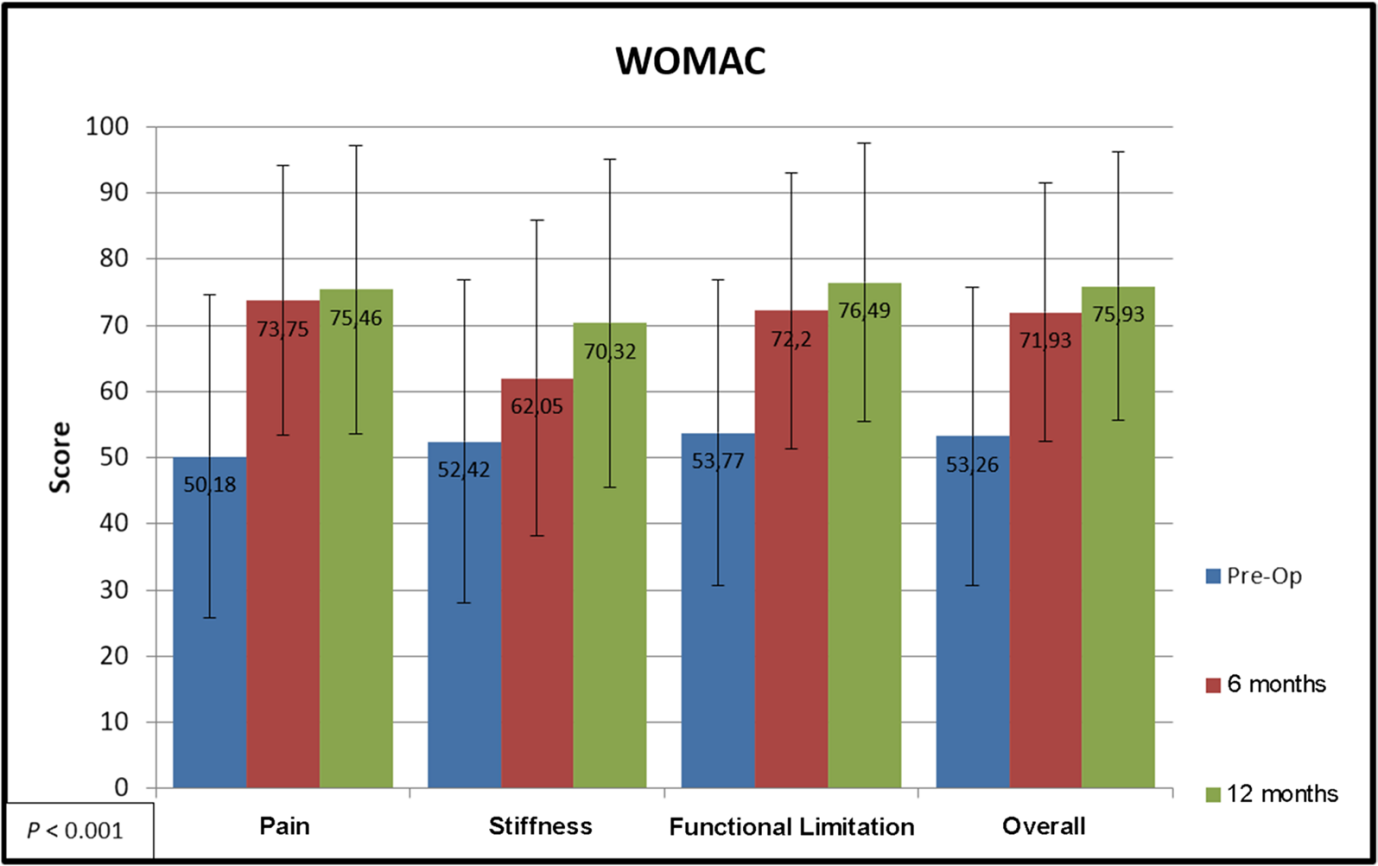
Descriptive clinical outcomes of articular joint repair: WOMAC score

All subjective WOMAC scores (pain, joint stiffness, and functional limitation) improved significantly from baseline after surgery (*P* < 0.001). Details are given in Fig. 4.

**Fig. 5:**
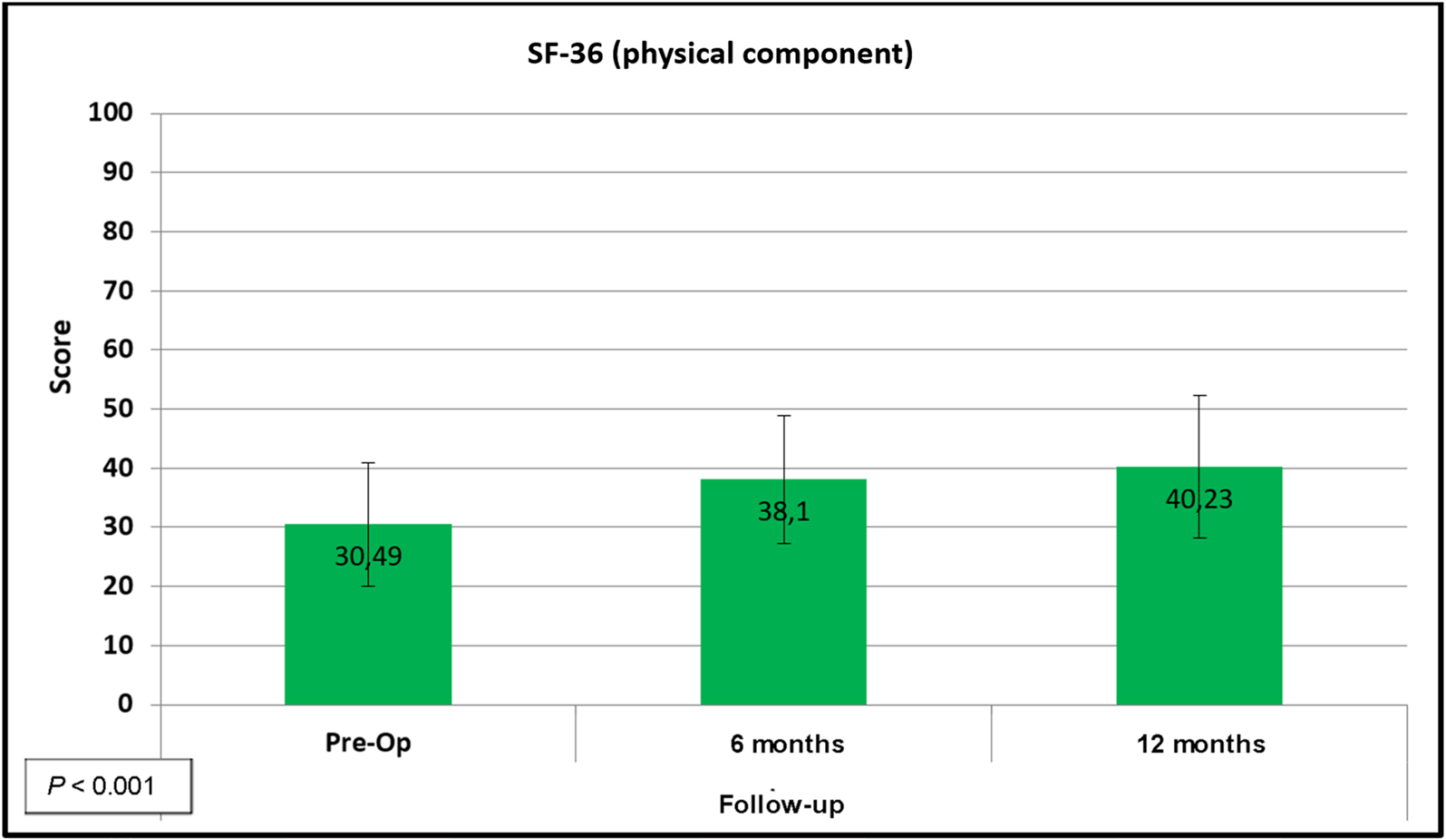
Descriptive clinical outcomes of articular joint repair: SF-36 score (physical domain)

Improvement in the physical component of the SF-36 quality-of-life score is shown in Fig. 5.

The mental component of the SF-36 quality-of-life score demonstrated improvement (*P* < 0.05), rising from 46.43 at baseline to 49.84 at 12 months.

When stratified into groups, age was the only relevant factor, with more favourable outcomes in patients under age 45 on the final IKDC score (from 37.8 to 65.0 in those younger than 45, versus 30.3 to 50.0 in those aged 45 or older; *P* = 0.010) and final total WOMAC score (from 60.8 to 84.4 in those younger than 45, versus 48.4 to 69.7 in those 45 or older; *P* = 0.022). Better results were also achieved by patients under age 45 on the VAS and SF-36 (physical and mental domains) at 12 months after surgery, but in these instruments, the difference was not statistically significant.

Likewise, there was no significant difference in IKDC, total WOMAC, VAS, or SF-36 (physical and mental) final scores when comparing patients with traumatic versus non-traumatic aetiology, previous surgery or no previous surgery, articular cartilage repair alone versus combined surgical procedures, or lesion location (patellofemoral versus femorotibial).

In five cases (7.7%), the procedure was considered to have failed. In one case, the implant came loose from the patella; it was removed, and the lesions (multifocal) were debrided. In two other cases, the patients subsequently required total knee replacement (one had a multifocal lesion, and the other, a 9-cm^2^ lesion). Two cases required second-look patellofemoral arthroplasty (both had multifocal lesions and had undergone combined procedures).

There were four complications during the follow-up period (6%), all in successful procedures: one deep venous thrombosis, resolved with conservative treatment only; two cases of arthrofibrosis, which improved with manipulation under anaesthesia; and a superficial skin infection treated with oral antibiotics.

## Discussion

The objective of articular cartilage repair is to provide long-lasting improvement of the patient’s quality of life. It is also important that the technique be readily available and be as harmless as possible. The arthroscopic debridement although gives some good results, the studies are limited, and the evidence is low [7]. On the other hand, the cultured chondrocyte implantation provides good repair capacity but has disadvantages such as high cost, limited availability, and the need for two procedures [8]. Biological interventions as mesenchymal and adipose expanded laboratory cells have some promising results, but some are experimental, and some studies question the durability and safety [9]. Salzmann et al. [6] demonstrated a procedure using a membrane seeded with autologous cartilage cells, which can solve the problems of cost and availability and be performed in a single stage.

Among the 64 repairs carried out in our sample, only once did the membrane come loose (1.5%); the other four cases of failure were likely due to progression of the underlying degenerative process, requiring conversion to arthroplasty (6.25%). Gomoll et al. [10], in a study of 101 patients with up to 1 year of follow-up, used fibrin glue and sutures along the periphery only when they felt the need for greater fixation, achieving a 5% reoperation rate due to hypertrophy or delamination.

In our study, VAS scores for pain decreased from 5.92 to 2.37 in the postoperative period (*P* < 0.001), demonstrating clinical improvement in this aspect at 12 months of follow-up. Siclari et al. [11], using the same microfracture scaffold combined with platelet-rich plasma and Smart Nails® (ConMed Linvatec Italy, Milan, Italy) and fibrin glue for fixation, also reported improvement in the Knee Injury and Osteoarthritis Outcome Score (KOOS) (from 54.1 to 93.5). Verdonk et al. [12] used the MaioRegen® three-layer scaffold (Fin-Ceramica Faenza SpA, Faenza, Italy) and likewise reported a reduction in VAS pain scores, from 63.1 to 22.7. Although pain may not be related to the articular cartilage lesion per se, it is interesting to note that it improves markedly after cartilage repair procedures are performed. This effect can be credited to restoration of the load-bearing property, to reduction of subchondral pressure by the microfractures, or to neovascularization of the lesion site, reducing inflammatory cytokine levels and thus relieving symptoms [13, 14].

We also observed improvements in IKDC scores at 12 months postoperatively (from 33.44 to 56.33, *P* < 0.001). Berruto et al. [15], using a three-layer matrix implanted through the “press-fit” technique in 49 patients, reported an increase in IKDC scores from 45.55 to 70.86 at 12 months. Delcogliano et al. [16] used the same technique and matrix in 19 patients (21 lesions) and demonstrated an approximate gain of 30 score points (35.7–67.7) in 12 months. Theoretically, a three-layer matrix mimics hyaline cartilage more closely. Even so, the clinical outcomes achieved are very similar and may be attributable to the use of bone perforations or autogenous grafts. In the present study, an approximate 23-point improvement in the mean IKDC score was observed, noting that patients had lower baseline (pre-operative) scores than in the case series of Berruto et al. [15] (45 points) and similar scores to those reported by Delcogliano et al. [16] (35.7 points). This may be attributed to the more advanced average age of our patients compared to the other series (44 years, versus 37 and 33 years respectively) [15, 16].

In our cohort, the total WOMAC score improved from 53.26 to 75.93 (*P* < 0.001) at 12 months post-operatively. Dhollander et al. [17] used allogeneic chondrocyte cell cultures from fresh tissues protected with periosteum. This procedure was performed on 21 patients aged 12–47 years, who obtained an average 42% improvement in WOMAC. For comparison, in our study, we observed a 42.6% increase in total WOMAC scores. Although part of the cases in the present study were of focal, trauma-related lesions, a higher percentage of patients had progressive pain, probably indicative of osteoarthritis rather than traumatic aetiology; the WOMAC score could be a better tool to assess these patients, since it is a scale designed for degenerative processes.

Few studies have demonstrated improvement in quality of life after cartilage repair. The SF-36 showed an improvement in its physical domain, from 30.49 at baseline to 40.23 post-operatively (*P* < 0.001). Gelber et al. [18] reported an improvement in SF-36 scores from 53.9 to 86.6. In our series, improvement was not as marked, which again may be explained by the higher average age of our patients (44 versus 36 years). Older patients tend to have a lower SF-36 score because they experience greater difficulty climbing stairs, kneeling, and doing other daily living activities. Cole et al. [19] reported an improvement in scores from 35.4 to 45.5 in the treatment of osteochondritis-related lesions. This more modest improvement, closer to ours, may be due to their patients having osteochondritis dissecans, which carries a less favourable prognosis. The normal range of the SF-36 physical domain score has been described as 49.7 ± 9.4 [20] in the Canadian population. Thus, it is clear that, despite improvement in patient quality of life, they are unlikely to reach the same level of quality of life experienced by the general population.

Knees with a cartilage lesion but no history of surgery and no comorbid pathologies, so-called “green knees”, exclude those most likely to have an unfavourable outcome. However, most patients (70% of cases) have combined lesions, or “red knees” [21, 22]. Martinčič et al. [23] followed 151 patients for an average of 10 years after knee cartilage repair. They found no statistically significant difference in outcomes between patients with and without a history of prior surgery. In our study, we chose not to exclude “red knees”, so as to ensure that our sample was representative of the patients most commonly seen in clinical practice. We found no significant difference when patients were stratified by traumatic versus non-traumatic aetiology, prior surgery, combined surgery, or lesion location.

Despite showing an improvement in scores, our patients aged > 45 years fared rather poorly compared to their younger counterparts. Turinetto et al. [24] demonstrated that advancing age decreases the potential for differentiation, immunomodulatory effect, and migration ability of pluripotential cells and chondrocytes, thus reducing the healing power of tissues. An alternative to improve this capacity would be to introduce cell growth factors such as SDF-1alpha [25], which has been demonstrated to increase recruitment of cartilage progenitor cells in laboratory studies or use the expanded mesenchymal cells—witch are showing encouraging results [26].

Overall, there were five cases of treatment failure (7.7%). Assessment showed that all patients needing conversion to total knee replacement had lesions that required greater healing potential and were over 45 years of age. The only patient whose implant came loose was also > 45 years old and had a large lesion, despite no history of trauma. Compared to those of other series, however, our failure rate was similar. Verdonk et al. [12] reported a 5.3% failure rate, and Delcogliano et al. [16], 2 out of 19 cases (10.5%).

## Conclusions

A combined technique consisting of microfractures, autogenous graft, a synthetic scaffold, transosseous sutures, and fibrin glue provides secure fixation for treatment of articular cartilage injuries of the knee. Patients experienced an average of 20 points of improvement in the IKDC and WOMAC scales and 10 points of improvement in the SF-36 score. Age greater than 45 years had a negative impact on outcomes.

## Data Availability

The dataset supporting the conclusions of this article is included within the article.

## Abbreviations

ANCOVA: Analysis of covariance
ANOVA: Analysis of variance
ATT: Anterior tibial tuberosity
BMI: Body mass index
ICRS: International Cartilage Repair Society
IKDC: International Knee Documentation Committee
SF-36: Medical Outcomes Short-Form Health Survey
VAS: Visual Analogue Scale
WOMAC: Western Ontario and McMaster Universities Arthritis Index
